# Understanding risk factors for musculoskeletal disorders in Iranian housewives: Development of a comprehensive health promotion behavior model

**DOI:** 10.1186/s12889-023-15518-w

**Published:** 2023-03-31

**Authors:** Samaneh Norouzi, Sedigheh Sadat Tavafian, Rosanna Cousins, Hamidreza Mokarami

**Affiliations:** 1 Department of Health Education, Faculty of Medical Sciences, Tarbiat Modares University, Tehran, Iran; 2 Department of Psychology, Liverpool Hope University, Liverpool, UK; 3 Department of Ergonomics, School of Health, Shiraz University of Medical Sciences, Shiraz, Iran

**Keywords:** Content analysis, Ergonomics, Health promotional intervention, MSDs, Theory of Triadic Influence, Quality of life

## Abstract

**Background:**

Chronic musculoskeletal problems are a major source of disability, reduced productivity and poor quality of life. Prevalence of musculoskeletal disorders among Iranian housewives is particularly high. Understanding how housework causes the injuries, mobility restrictions and pain associated with musculoskeletal disorders is vital to developing health promotion behavior models to support intervention. The aim of this qualitative study was to explore the experiences of housewives with musculoskeletal disorders and, according to the risk factors identified, to develop a comprehensive behavior change framework to facilitate implementing a musculoskeletal health-promoting training intervention for women.

**Methods:**

Twenty-four in-depth interviews were conducted with Iranian housewives aged 20–65 years experiencing musculoskeletal pain over a 13-month period from September 2020 to October 2021. The conventional content analysis approach was used to interrogate the data. The transcript of each interview was considered a unit of analysis, and data analysis was performed using MAXQDA2018 software.

**Results:**

The analysis of the interview data provided 1432 meaning units. From these codes, a conceptual framework was developed. This comprehensive model is comprised of 24 subcategories, eight categories and three themes: Individual and social predictors of MSDs and their control, Risk factors for MSDs, and Prevention and treatment of MSDs. Altogether the developed conceptual framework specified the multiple risk factors for MSDs in housewives. The findings were aligned to various health promotion models, and it was seen that ecological models, especially the Theory of Triadic Influence, can be very helpful as a supportive roadmap to implementing multilateral interventions to improve the quality of life of housewives.

**Conclusions:**

This study developed an evidence based comprehensive model that identifies the individual, psychosocial, and cultural factors that influence the status of MSDs in women’s domestic work to support the development and implementation of effective ergonomic interventions to manage potentials for MSDs.

**Supplementary Information:**

The online version contains supplementary material available at 10.1186/s12889-023-15518-w.

## Introduction

Musculoskeletal disorders (MSDs) caused by work are a significant cause of disability which in turn impacts on workability, and quality of life. In recent years, there has been an increase in ergonomic research to investigate the causes of the various types of MSDs to support effective intervention. Musculoskeletal disorders are common pains that affect muscles, bones, joints, ligaments, and tendons [1]. MSDs affect about one in ten people in the general population, with an overall prevalence estimated at 11.2% in women and 7.2% in men [2]. In their systematic review of musculoskeletal pain prevalence in the general population, Andrews et al. [2] note that studies examining both men and women consistently show that women experience higher levels of musculoskeletal pain than men. This assertion is supported by studies that have specifically examined gender differences in MSDs, and shown that women are at higher risk than men [3]. The consensus is that the primary reason for those differences is the anthropometric and physiological differences between men and women [4].

In addition to formal workplace risks, women are also at great risk for MSDs while doing housework tasks [5]. In many cultures, housework is an esteemed role for women, and the majority of women are satisfied with the gendered division of domestic labor [6]. For example, in India two studies have indicated a very high prevalence of musculoskeletal pain: 41% and 83%, with the latter study suggesting that more than 50% of housewives are disabled by MSDs [7, 8]. Similarly, in Iran, prevalence studies indicate that an estimated 53% of housewives have MSDs [9, 10]. This equates to many millions of women with MSDs in Iran, as the proportion of women over 15 years in employment is estimated to be 6% [11]. Although women have equal access to higher education in Iran, the dominant economic family model remains the male breadwinner model.

Ultimately, research indicates that MSDs are chronic physiological issues that interfere with people's lives and adversely affect their daily activities, physical and mental health, family and social relationships, and their work [12]. This finding extends to many job roles, and the issues often have a significant adverse impact on the quality of life of individuals and affect all physical and mental components of their lives [13]. It remains unclear the extent to which behaviors associated with housework contribute to MSDs, which is important to understand risk factors to be able to develop health promotion interventions to support better health in full-time housewives. For instance, in Iran there are cultural reasons for keeping a clean house, and home making is held in high esteem. There are long established beliefs that a house must be clean at all times, and before New Year, there is an extensive whole house clean, that includes polishing every corner and every item in the house. This is a publicized national celebration. The importance of this ritual can be related to a belief that an unclean house and living environment would cause health problems for the family. Thus, housework provides the basis for a healthy life for the family.

Previous research has shown that MSDs are a multifactorial phenomenon affected by various risk factors [14]. These factors can generally be divided into biomechanical, psychosocial, sociocultural, and individual risk factors [12, 15]. Biomechanical risk factors include repetitive movements, carrying heavy objects, and awkward bodily positions [1]. Psychosocial risk factors include psychological distress, lack of support from others, and lack of decision-making power [16]. Social factors include culture, beliefs, and cultural beliefs [12, 17], and individual factors include issues related to lifestyle, age, gender, body mass index, and living conditions [18]. Given the influence of multiple risk factors on the incidence of MSDs, implementing any preventive and control programs and measures requires the simultaneous identification and control of a set of risk factors [1].

Housekeeping is one of the most demanding jobs for women involving alteration of many normal biomechanical parameters during the course of executing the activities involved, and a high level of physical burden [5, 19]. There are many workstations for women at home, and they perform a variety of activities in these workstations. The tasks include cooking, washing, shopping, and caring for family members and children, all of which require considerable physical, emotional, and mental activity [20]. Although the cited evidence confirms that many housewives suffer from a broad range of MSDs caused by numerous risk factors, it is still unclear how they deal with their health and how their behaviors and beliefs improve their health. To advance the situation, it is essential to identify behaviors and actions with positive and negative effects on housewives' musculoskeletal health. Despite the significant risks of MSDs among housewives, few studies have addressed these risk factors comprehensively. It is not possible to measure most of the experiences, perceptions, beliefs, and characteristics that are involved in housework. In addition, due to the influence of social and cultural factors, values, and traditions in housekeeping activities, it has become necessary to examine housewives’ experiences and views in different cultures. Thus, to develop and implement comprehensive and purposeful training interventions and measures, it is essential to have a clear understanding of the experiences of housewives it is necessary to use qualitative methods and in-depth interviews with a representative sample of housewives. To our knowledge, no effort has yet been taken in this regard. Furthermore, more desirable outcomes can be achieved through developing educational programs in line with well-established theories and models [21]. Theories and models can serve as educational roadmaps. They provide guidelines for educational review and diagnosis, educational planning, and interventional design, and facilitate evaluating educational outcomes [22]. To this end, a framework was developed in this qualitative study to support the design of a health promotion model tailored to the needs of housewives. Accordingly, this study followed two objectives:

1. To provide a comprehensive understanding of the experiences of housewives in terms of the multiple risk factors for MSDs.
2. To develop a health promotion model in line with the conceptual framework developed in this qualitative study to be able to implement goal-directed intervention programs and measures.

## Methods

### Participants

This qualitative study was conducted in Akbarabad-Kavar, which is located 50 km from Shiraz, the capital of the Fars province in Southern Iran. A pre-COVID census estimated the population of the town to be about 8,000 adults. The population was typical of the middle eastern culture: family income was provided by working husbands, and wives worked in the home. Purposeful sampling was used to recruit full-time housewives aged 20–65 years with a diagnosed MSD disorder. To meet these inclusion criteria, the study was advertised at a specialist musculoskeletal disorders treatment clinic for women. The exclusion criterion was having any job other than housekeeping tasks. Accordingly, women who gave informed consent took part in the study and were interviewed.

### Procedure

The interviews were all conducted by the first author (SN) in the participants' home in a quiet private room between September 2020 and October 2021. The interviewer was female, but not an insider researcher as at the time of the study not married and with no history of musculoskeletal pain. Nevertheless, potentials for bias were carefully considered in the research team, and as a PhD student, the interviewer sought to bracket any pre-conceptions of influences in all aspects of the study with supervisors. As a part of ensuring reliable data collection, the semi-structured interview guide developed to steer all the interviewers was collaboratively developed with all authors, and it followed the published principles and practices of Olson [23]. The interviews lasted between 30–100 min, according to the housewives’ experience and ability to articulate it. All interviews were audio-recorded.

After receiving the participants’ demographic information, each interview began with participants being were asked to share their experiences of the tasks they performed during a normal day in terms of housework and childcare. This was then extended to go through all seven days. Prompts were made, as required, to include detailed information regarding support that may be given by others, and to ascertain which household tasks were not being done, and why. The interview would proceed to investigate biomechanical aspects of the housework that participants were doing, to understand how tasks were executed, pains that were experienced during task performance, and which tasks were done differently since experiencing pain, and how, and the benefit or otherwise of changes. Then information was collected on psychosocial aspects of the housework including seeking support, and support given; coping with pains and responding to that in terms of emotions and practical responses; social life and changes, as well as impact on quality of life of pain associated with MSDs.

A summary of the participant's responses was presented to them after each question in the interview. In cases where there was ambiguity or inaccuracy in the answers (e.g. sometimes), the interviewer asked the participants to give more information to clarify their statements, or to provide an example. This served to further enrich the data.

### Data analysis

Data analysis was performed continuously and simultaneously with data collection using the conventional content analysis approach [24]. Two of the researchers (SN, HM) first listened to the audio files of the interviews several times then the recordings were transcribed. The transcripts were then read several times to check if their content was the same as the content of the audio file. Then, the audio files were deleted. The unit of analysis in this study was the written transcript of each interview, and the data were analyzed using MAXQDA2018 software to identify risk factors for musculoskeletal disorders (MSDs) reported by the participants. The participants' statements that contained significant information were selected as meaning units to extract the initial codes. An iterative approach was used to classify the extracted codes starting with those identified in the first interview. Each new code from subsequent interviews was compared with the previously identified codes and was placed into a relevant category. The research team reviewed and compared the extracted categories several times to identify further categories and codes. Data collection continued until the collected data were saturated.

### Trustworthiness

The credibility, dependability, transferability of the data and findings were checked using the method proposed by Graneheim and Lundman [24]. Credibility was established through long-term engagement in data collection (thirteen months) and member checking (by asking the participants to confirm the accuracy of the contents of the transcripts, and to revise them if necessary). The data analysis process was conducted by a research team (authors) who are experts in different fields (ergonomics, health promotion, and human behavior and psychology) by reviewing the extracted codes and related categories. After 21 interviews, no new code or information appeared indicating that the data were saturated. However, to further validate the findings, additional interviews were conducted with three more participants. The codes were revised if necessary. The dependability of the data was established using a semi-structured interview guide for all interviews and classification during the data analysis process.

## Results

A total of 24 women gave informed consent and took part in the interviews. Participant’s age ranged between 24 to 65 years, and the average age was 43.29 ± 10.02 years. They had been working as housewives for an average of 24.41 ± 12.41 years. Regarding marital status, 22 women were living with their husbands, and two were widows. All participants had children, with the majority having three children living at home. Regarding education, 87.5% housewives participants were educated to the level of high school diploma, one was illiterate and two had a university degree. The cohort as a whole was overweight: average BMI was 27.07 ± 4.09, although the BMI range was 19.02 – 36.5. The social economic status of 18 housewives was moderate (75%), five were impoverished, and one was classified as good.

To achieve a comprehensive understanding of the experiences of housewives in terms of the multiple predictors of MSDs, a conventional content analysis [24] of the interview data was undertaken. A total of 1432 meaning units were extracted from the 24 interviews conducted. From these codes, a conceptual framework was developed based on 24 subcategories, eight categories and three themes: Individual and social predictors of MSDs and their control, Risk factors for MSDs, and Prevention and treatment of MSDs (see Table 1). We provide illustrative quotes from selected participants for each subcategory according to category in the theme findings below. The quotations reported in this manuscript were translated from Persian to English in the draft stage. Each is accompanied by a brief description of the woman’s age to provide context. It should be emphasized that many of the predictors of MSDs that emerged from our data were issues that impacted many of the participants. That is, the majority were endorsed in some way by most of the participants.

**Table 1:** Themes, categories, subcategories, and sample codes from the content analysis

Sample codes	Subcategories	Categories	Themes
Individual and Social Predictors of musculoskeletal disorders and their control	Personality and Cognitive Functions	Adaptation to pain	* Enduring the pain * Coping with pain * Hiding pain from others * Ignore pain under any conditions
		Perfectionism in doing tasks	* Perfectionism in doing chores * Excessive obsession with house cleaning * Sensitivity to house cleaning * Sharing home tasks with others would lower standards
		Sacrifice for the sake of the family	* Carrying out home tasks alone due to compassion * Sacrifice own time and effort for family members and tolerate family demands * Not asking for help from family to do housework
		Personal beliefs	* Inevitability of musculoskeletal problems * Lack of belief in the effectiveness of medication * Disbelief in seeing a doctor
	Cultural factors	Cultural taboo	* Doing tasks despite pain (fear of being incapable( * Do things beyond physical strength (to look strong) * Fear of being called useless * Hiding the period cycle
		Normative beliefs	* Women are obliged to do all the housework * Competitive atmosphere between women in doing housework * High expectations of others from the housewife in performing duties
	Conditions of a person’s life	Family climate	* Family issues and unpredictable events * Death of loved ones and neurological problems that follow * Raising children in the absence of a spouse
		Economic conditions	* Use of defective equipment due to economic problems * Concerns and nervousness due to economic problems * Economic inability to see a doctor
Risk factors associated with musculoskeletal disorders	Psychosocial risk factors	Social support	* Lack of support from husband in doing housework * Lack of understanding of the difficulties of housework from husband * Lack of support from other family members in doing housework * Insignificance of family members towards the housewife
		Psychosocial stress	* Increase of stress as a result of feeling the pain * Mental distress and stress * Harassment and stress from others
		Stress performing tasks	*Increase of stress as a result of delay getting housework completed * The stress of not doing housework efficiently * Feelings of despair and hopelessness as a result of inefficient tasks * Stress caused by the high amount of housework demands
		Concerns related to children	* Concerns about children's future work * Concerns about children's health * Concerns about the safety and well-being of children
	Physical risk factors	Age-related problems	* Increase of the skeletal pain * Physiological changes in the body * Weakening of muscles and bone tissue * Decrease in physical capacity
		Femininity problems	* Multiple deliveries and childbearing * Long / painful menstrual cycle * Cesarean delivery
		Chronic disorders	* Obesity * Mental illness * Diabetes
	Biomechanical Risk factors	Awkward posture during housework tasks	* Twisting * Bending at the waist * Forward bending * Extended reaching
		Static postures during housework tasks	* Prolonged standing while cooking * Prolonged standing while washing dishes * Kneeling for long periods
		Perform tasks continuously and without interruption	* Performing multiple housework tasks without interruption * Hours of cooking without taking a rest break * Prolonged cleaning without taking a rest break
		High workload and Forceful exertions	* Lifting heavy objects * Pushing heavy objects * Vacuum cleaner with heavy handle
		Poor ergonomic house design	* Improper height of the sink * Improper stove height * High height kitchen counter * Traditional toilet design
Prevention and treatment of musculoskeletal disorders	Ergonomic solutions	Doing exercises	* Warm-up body before starting work * Stretching exercises
		Principles of ergonomics	* Work in a good posture * Take a short break while performing tasks * Alternate washing between the right and left hands
	Pain relief strategies	Non-invasive pain relief strategies	* Going for a walk * Talking to others (to forget pain) * Worship * Reading the Quran and praying
		Treatment strategies	* Herbal drinks * Pain relief creams * Taking medication * Physiotherapy solutions

### Individual and social predictors of MSDs and their control

The three categories in this theme were recognized as important predictors of MSDs and pain control by the participants as a whole.

The ‘personality and cognitive functions’ category comprised ‘adaptation to pain’ (*I have so many pains that I have got used to them. There’s no treatment for back pain and knee pain so I have to get along with them*. 41-year-old woman), ‘perfectionism in doing tasks’ (*I’m not satisfied with the housework done by others and my work must be done by myself perfectly*. 27-year-old woman), ‘sacrifice for the sake of the family’ (*I do the housework alone to provide more welfare for my husband and children*. 31-year-old woman), and ‘personal beliefs’ (*I cannot help it. Housework is such that you will inevitably get musculoskeletal problems.* 26-year-old woman).

‘Cultural factors’ contained two subcategories – ‘normative beliefs’ (*A housewife should not refuse to perform her duties because it is a sign of her weakness*. 47-year-old woman) and ‘cultural taboos’ (*I get very annoyed during menstruation. Sometimes I’ve had guests and despite the menstrual cramps, I was forced to do housework. It’s taboo for someone to be told that I have my period of menstruation*. 29-year-old woman).

‘Conditions of a person’s life’ included ‘family climate’ (*My husband’s workplace is very far from here. He may come home once a month. This means I have all the responsibility for taking care of the children and the housework*. 52-year-old woman) and ‘economic conditions’ (*I’m always worried about the economic status of the family*. 36-year-old woman).

### Risk factors associated with MSDs

The ‘risk factors associated with MSDs’ theme was made up of three main categories including ‘psychosocial risk factors’, ‘physical risk factors’, and ‘biomechanical risk factors’, and twelve subcategories.

‘Psychosocial risk factors’ included ‘social support’ (*My husband is at work every day. He doesn’t help with household chores, and he does not do so when he’s at home*. 35-year-old woman), ‘psychosocial stress’ (*People around us cause much stress into our lives because others interfere a lot in our lives. This makes me nervous and stressed, leading to more pain in my neck*. 48-year-old woman), ‘stress performing tasks’ *(I get very stressed and restless when I fail to do the housework, so if I want to die, I have to do my housework first and then I die. It’s terrible but I can’t help it. I get nervous and can’t relax when I have some household chores to do*. 42-year-old woman), and ‘concerns related to children’ (*I think a lot about the future. I wonder who will take care of my children if something bad happens to me. I will think a lot about these things and cry*. 37-year-old woman).

The ‘physical risk factors’ category consisted of ‘age-related problems’ (*When comparing now to ten years ago, I had no problem then. I did not even have a headache, but now I have pain in all parts of my body*. 42-year-old woman), ‘femininity problems’ (*I had three miscarriages. After that, my back pain worsened*. 55-year-old woman), and ‘chronic disorders’ (*I have put on a lot of weight and it has affected me a lot. I did not have any of these pains before, when I was slimmer. I get into trouble when trying to do anything because I’m overweight. I wish I could control my weight to have less pain in my back and feet*. 45-year-old woman).

Biomechanical risk factors whilst doing housework were also important issues highlighted by most of the participants. These risk factors were related to ‘awkward posture during housework tasks’ (*When sweeping the floor, I have to bend my back too much and then I get a strange back pain*. 35-year-old woman), ‘performing tasks continuously and without interruption’ (*When I’m washing the dishes, I’m also cooking. Immediately after that I do dusting. This is my job every day*. 57-year-old woman), a ‘high workload and forceful exertions’ (*I have to carry heavy objects alone*. 42-year-old woman), and ‘improper house design’ (poor ergonomic design) (*The height of the sink is very short and I constantly have to bend my back and neck*. 31-year-old woman).

### Prevention and treatment of MSDs

In addition to being aware of risk factors for MSDs, the participants stated that they used preventative and treatment strategies to reduce musculoskeletal pain. These strategies were distinguished in two main categories – ‘ergonomic solutions’ and ‘pain relief strategies’. ‘Ergonomic solutions’ were subcategorized into ‘doing exercise’ *(My pain is much better as soon as I started doing exercise compared to the time I was not exercising. I got to a better mood and my back pain got much better*. 45-year-old woman) and following the principles of ergonomics (*I try not to lift heavy objects alone. I must get help from someone because it will hurt me later.* 42-year-old woman).

The participants in this study stated that they used various psychological, religious, and treatment coping strategies to relieve their pain. The ‘pain relief strategies’ category were included ‘non-invasive pain-relief strategies’ (*When I have pain in my body, I say prayers and read the Quran. You don't know how relaxing it is and how much it is effective in relieving pain. If we learn to get help from God, he will alleviate our pains without the need for any doctor and medicine*. 24-year-old woman) and ‘treatment strategies’ (*I’m always nervous. Chamomile is very effective and makes me feel relaxed. When I have pain in my back, I use fennel oil and eggs, and I feel much better. I think these remedies are much better than chemical drugs prescribed by doctors*. 60-year-old woman).

### Development of a participatory health promotion model

**Fig. 1:**
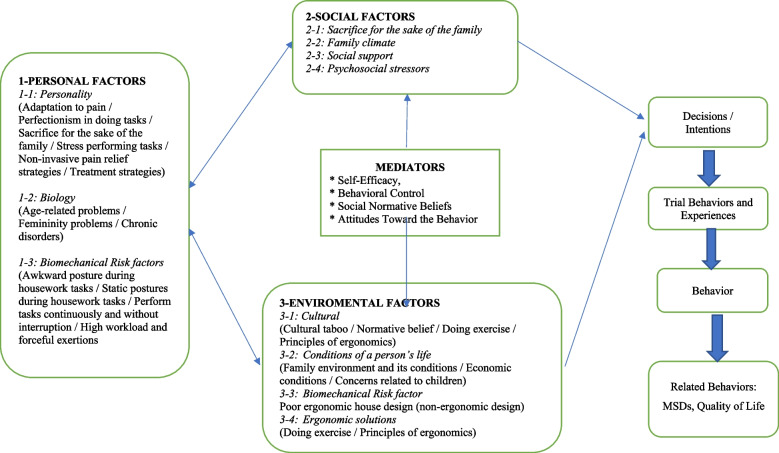
A comprehensive health promotion perspective on risk factors for MSDs

The themes, categories and subcategories that emerged from the content analysis of the interview data were used to develop a comprehensive health promotion model that will act as a roadmap for designing behavior change educational interventions [22] for managing MSDs. The interview data showed that a variety of individual, psychosocial, and cultural factors can act as predictors of MSDs. The Theory of Triadic Influence (TTI), as an ecological model [25], was recognized as being a particularly suitable conceptual framework for using the results to intervene. The TTI is an integrative theory for promoting health behaviors that uses a matrix of three (intrapersonal, interpersonal, and peripheral) impact streams and three (ultimate, distal, and proximal) impact levels [26]. The TTI model indicates that some variables (e.g. intent) will have a direct effect on behavior, and these are considered causal factors. Some variables (e.g. satisfaction of others) have effects that are mediated by other variables that are determined by individual differences in attitudes and capabilities (e.g. social normative beliefs) and that these are more distal in terms of inducing target behaviors. In addition, there are other variables that are relatively stable, but not readily amenable to change by individuals (e.g. ethnic culture, neighborhood poverty), which represent the underlying or ultimate causes of behavior [25]. The conformity of the conceptual framework extracted from the qualitative study with each of the streams of the TTI model is presented in Fig. 1, and a brief explanation is provided in Table 2.

**Table 2:** The theory of triadic influence applied to the qualitative data analysis

Levels of influence	Definitions of the types of influence		
	Intrapersonal (Biology/personality)	Interpersonal (Social)	Environmental Broader
Ultimate	Although the personality and biological characteristics of housewives are unchangeable and uncontrollable, they induce some intrinsic motivations for developing MSDs (such as denial of pain, lack of stress management, perfectionism in performing daily tasks and self-treatment; the presence of chronic diseases, age-related changes, gynecological problems, and multiple pregnancies)	Characteristics of people who can support housewives to prevent developing MSDs. These characteristics (e.g. values and social norms) are beyond individual control but expose them to MSDs	Social and environmental risk factors for MSDs, such as cultural taboos, poor ergonomic house design, limited facilities at home
Distal	Emotional states and general behavior skills that induce MSDs in housewives or contribute to developing MSDs, such as low self-efficacy in managing psychosocial stress and coping with pain and perfectionism	Emotional states and attitudes of housewives that exacerbate MSDs, such as sacrifice for the sake of others, the family climate, and the resulting psychosocial distress	Emotional states that a housewife experiences as a result of their living environment, such as the values that govern society
Proximal	Belief in a person's ability to reduce MSDs, e.g. increasing self-efficacy in controlling perfectionism, preventive measures, stress management, and self-medication	Belief in the normative nature of MSDs, such as not seeking help from others to help with housework tasks, and controlling psychosocial distress	Belief in the ability to reduce MSDs, (e.g. by making changes in the work or way of working to reduce pain)

The three streams influencing behaviors that can lead to MSDs among housewives are: (1) *Intrapersonal (biology/personality)*: This type of influence reflects how the personality and biological characteristics of housewives impacts upon their ability to control musculoskeletal pain. Personality traits are predominantly intrapersonal but they can also be influenced by some external factors [27]. (2) *Interpersonal (social)*: This stream reflects the extent to which interpersonal or social situations and support, and psychosocial pressures exacerbate MSDs in housewives [25, 26]. (3) *Environmental (sociocultural)*: These are factors that reflect the characteristics of a broad cultural environment, and general social values that contribute to the development of MSDs and attitudes toward a behavior [26].

## Discussion

The aim of this study was to explore the experiences of housewives with MSDs and to develop a comprehensive behavior change framework or model for implementing effective health promotion educational interventions based on the needs of participants. The three themes extracted from the qualitative study provided valuable insight into the working conditions of housewives, and provided the basis for the development of the health promotion model we have proposed. The first theme covered individual and social predictors of MSDs. The participants typically believed that as MSDs were incurable and preventable, and that they should be avoided or at least ameliorated by adapting the circumstances of their work. Nevertheless, evidence of failure to pay attention to personal health, and underestimating the reasons for their pain, could be due to both a lack of awareness of the causes of MSDs, and ways to prevent them. These are aspects that could be a part of an educational intervention, using the model developed, to remove the seeming inevitability of MSDs. It may be that women have naturally learned to live with pain and tend to consider it something normal [28], however, there was also evidence of perfectionism in performing household tasks, and sacrifice for the sake of the family, both without paying attention to one’s health due to special individual beliefs that there were other individual factors affecting the incidence of MSDs. Doing household chores perfectly and without asking for help from others due to feelings of duty and personal commitment takes a lot of energy and time and will lead to physical and mental burnout in the long run [29, 30]. Furthermore, the cultural environment and normative beliefs formed over time exacerbate this issue. These may force a woman to do tasks that are beyond her physical ability to avoid the negative consequences of being considered a weak person, or incompetent, by those around her. Such beliefs are common and have been an issue among different cultures since ancient times. Critically, these beliefs hinder the use of logical and scientific strategies and preventive principles for maintaining and promoting health. Moreover, obsessive thoughts and cognitive beliefs prevent the expression of pain by women and play a vital role in the persistence of MSDs [28]. These issues can be addressed using an intervention approach to managing MSDs.

The second theme identified in this study covered risk factors associated with MSDs. A majority of the housewives pointed out the prominent role of biomechanical risk factors, suggesting the high prevalence of MSDs among housewives is due to the nature of household tasks such as preparing food, washing clothes, cooking, and cleaning. As previously reported [31, 32], the biomechanical demands of housework are such that women are forced to work in poor and awkward postures that can damage different parts of their bodies. Household chores include carrying heavy objects, repetitive and continuous movements, and frequent and uninterrupted bending [32]. The housewives reported that they often had to perform multiple tasks without taking adequate rest due to lack of time. Thus, these tasks can contribute to the development of MSDs in women [31]. Education regarding posture, movement and biomechanical stressors can serve to manage these risk factors,

As people get older, it is assumed that they face more physical vulnerability to MSDs. In this study, some participants attributed their pain to age when in fact, they were not yet of the age that one would obviously expect them to have age-related MSDs. Indeed, we had participants who asserted that their pain and decrease in mobility was related to getting older, when in their 30 s and 40 s. Age and MSDs have a complex relationship. Longitudinal survey data from a substantial Finnish study [33] indicated that there were different risk factors for MSDs at times of working life – and critically, in this study the biggest risk for MSDs from repetitive movements and awkward postures was found for those aged 36–49. Ultimately, the relationship of age and MSDs in that study was not statistically significant, and the authors concluded that an ergonomic, task-related approach is required to manage MSDs. Thus, we too consider that age is a misunderstood factor in the onset of MSDs, but this does not mean that there are not particular vulnerabilities at particular times of life. For example, multiple pregnancies and deliveries, can lead to MSDs [34]. The majority of participants in this study had three or more children, thus, in addition to many responsibilities of housework, at this time of life their parenting role imposed a substantial workload on them. Altogether, there is compelling evidence that women need to be taught how they can comply with ergonomic principles of care to reduce MSDs at all times of life, and they are a threat to healthy aging, rather than an outcome of aging [29].

Psychosocial risk factors such as poor social support for housewives and psychosocial distress were other risk factors for MSDs identified in this study. The participants reported that the performance of various household tasks was problematic, and they needed empathy and emotional support from their family members. They wanted to be supported, understood, and appreciated. Perceived support from others is directly related to reducing the severity of pain [35]. Thus, support from family members in household chores can reduce the high levels of psychosocial stress experienced by housewives and should be considered in any model to promote health.

The housewives in this study acknowledged the role of ergonomics in reducing the incidence of MSDs. However, for the reasons mentioned above, they did not follow these principles. Various studies have emphasized the effect of ergonomic principles such as stretching and strengthening exercises, maintaining good posture while performing tasks, and adequate rest while performing tasks to prevent the occurrence of MSDs [36]. Studies have also highlighted the effect of stress reduction strategies and promotion of mental and spiritual health on the promotion of physical and musculoskeletal health [37]. Since housewives face multiple physical and mental risks, comprehensive intervention programs developed to promote their musculoskeletal health should cover the various biomechanical risks and seek to promote mental and spiritual health in housewives, whilst considering mediating factors where relevant to ensure maximum, sustainable health benefits. Participants emphasized the effectiveness of biomechanical solutions in reducing pain associated with MSDs. Most musculoskeletal problems can be effectively managed through regular medical visits and using medical services. The housewives reported that although a visit to their doctor was effective in reducing their musculoskeletal pain, after the end of the medication period, the pain intensified again. This problem can be attributed to the housewives’ failure to fully comply with recommendations regarding exercise therapies, as there is significant evidence that musculoskeletal pain can be reduced by adherence to medical instructions [38]. Nevertheless, we would argue that there is scope to support adherence through education in a purposeful participatory health intervention.

### Explanation of the conceptual framework

As noted earlier, MSDs are multifactorial issues affected by various risk factors. Thus, their treatment requires a comprehensive approach to implementing intervention programs and measures. The conceptual framework developed in this qualitative study also confirmed this issue. For this reason, among various health promotion models, ecological models had the best compliance with the conceptual framework proposed in this study. Ecological models are developed based on the presumption that individual behavior is the outcome of a dynamic interaction with the social environment, which includes different levels of impact on health behaviors including intrapersonal / individual factors, interpersonal institutional and organizational factors, community factors, and public policy factors [39, 40]. Accordingly, the TTI model can provide an educational roadmap, and the necessary instructions for educational review and diagnosis, educational planning, and design of musculoskeletal health promotion interventions for housewives. TTI is an ecologically accurate approach to changing health behaviors and suggests that focusing more on the distal and ultimate levels has greater and more lasting effects than traditional approaches that focus on the proximal level. According to the TTI model [25], individual behaviors are influenced by the interaction of personality / biological (intrapersonal self-efficacy), social (interpersonal social normative beliefs), and cultural factors (sociocultural attitudes). Each stream of the model suggests several approaches to changing health behaviors, and these should be incorporated into a comprehensive preventative model to reduce the occurrence of MSDs.

The personality / biological stream shows that there are two ways that health professionals can improve health-related self-efficacy among housewives. First, they can teach self-regulatory and self-management skills to housewives who have poor self-concept or poor self-control to express pain. Otherwise, they are at risk for MSDs. Second, they can improve women's skills and self-confidence to know how to deal with situations that enhance the risk of musculoskeletal problems. For example, health professionals can use their abilities to help housewives prevent the progression and chronicity of musculoskeletal by expressing their pain.

The social stream shows that health professionals can provide prominent role models to support housewives to adopt healthy lifestyles [41]. They can also try to change the behavior of relatives and family members who actions are involved in the behavioral health risk patterns for MSDs [42]. The TTI can also help reduce social pressure by correcting distorted perceptions (knowledge or cognitions) about how people’s behaviors can lead to MSDs and their motivation to follow or satisfy people [25].

The cultural-environmental stream suggests a broad range of ways that can be used by health professionals to improve the musculoskeletal health of housewives. The most obvious way is to provide information and knowledge about the health-related consequences to housewives. Knowledge is the key to promoting health, and the key to changing health-related behaviors is to provide people with new health information. For example, if housewives are aware of the risks of developing MSDs and their consequences for life, then they can logically decide to refrain from engaging in behaviors that lead to MSDs. The presumption that knowledge and awareness are power underlies many health promotion efforts and historically, it is the first approach to adopt when seeking health behavior change [43].

It remains, however, that the TTI reminds us that the most effective interventions are probably those that target a wide range of these factors. For example, education about information, values, and decision-making may not be enough to change behavior, especially if people are exposed to multiple conflicting messages. Health professionals may perform a more effective role by participating in the sociocultural environment in which people live. TTI improves curricula that address mediating social skills, self-efficacy, attitudes and social norms as well as information. However, given that behaviors are induced in the social stream in families and society and the cultural-environmental stream at the sociocultural level, it is necessary to educate whole families and communities through cultural-social and environmental interventions, programs, or campaigns.

A key limitation of this study was its reliance of recall in data collection. Nevertheless, collecting data in the place in which housework practices, and thus the associated learning and memory took place should support more accuracy in recall [44] than undertaking the interviews in a neutral place unfamiliar to all participants. Whilst all participants in this study were Iranian housewives, since a majority of household tasks are commonly performed by women in most nations, our findings are at least partially relevant to the entire community of housewives in all parts of the world.

## Conclusions

This study has provided evidence of multiple factors that can contribute to the development of MSDs in housewives. These include: Ignorance of causes of musculoskeletal pain and poor self-efficacy, Lack of awareness and social support for housewives, and Lack of environmental facilities. Effective ergonomic interventions can be implemented through frameworks and models that address various individual, psychosocial, and cultural factors. In this regard, the Theory of Triadic Influence can be very helpful in identifying and implementing a comprehensive ergonomic intervention for preventing MSDs and ameliorating further pain and deterioration. Future studies are needed to assess the efficacy of the proposed comprehensive health promotion model that emerged from the data. Further studies can also examine MSDs in housewives with a broader range of demographic characteristics.

### Supplementary Information

**Additional file 1:** Interview guide

## Data Availability

The datasets generated and analysed during the current study are not publicly available due to confidentiality issues. An anonymised version of the raw data available from the corresponding author on reasonable request.

## Abbreviations

MSD: Musculoskeletal disorders
TTI: Theory of triadic influence
