# Supplementary material for: Understanding risk factors for musculoskeletal disorders in Iranian housewives: Development of a comprehensive health promotion behavior model
Source: BMC Public Health. 2023 Mar 31;23:617. doi: 10.1186/s12889-023-15518-w (PMC10064530; doi:10.1186/s12889-023-15518-w)
Supplement: Supplementary file 1 — Additional file 1: Interview guide [file 12889_2023_15518_MOESM1_ESM.docx]

**Interview guide**

This interview guide is designed to be flexible. It is essentially a grounded approach to understand pain and distress associated with the work of a housewife. It is an outline of the topics and issues that the interviewer wants to be certain to cover in the interview, without constraining the flow of conversation and explanation provided by the participant.

Although I have had to put points down in some semblance of order, this should not be seen as prescriptive in the interview process. Ultimately the goal is to provide information to support good intervention, and this means getting detail without leading or bias.

The guide is also designed to work as a tool to enable the participant to reach a point where they can provide a thoughtful answer to the final question after discussing the various aspects of their situation as a whole.

The usefulness of the guide will emerge with pilot testing, or a review after two or three interviews have been undertaken. If not comprehensive, or capturing (only) the constructs of interest, then we should edit. We want the outcome of the huge effort of the Phase 1 stage to provide the best support for the model and intervention we are designing.

*Introduction*

The interviewer introduces themselves and confirms what was on the Participant Information Sheet (associated with Informed Consent at the recruitment stage). The interviewer will encourage the participant to speak candidly and fully explain their situation in detail. There are not wrong answers; the goal is to understand their work at home and the physical pain and psychological distress associated with it. Permission to record the interview is confirmed, and assurance of feedback is provided (as part of the member checking process). The participant should be given the opportunity to ask questions before continuing.

*Primary question*

“My questions today will focus on understanding all about the pain in housewives takes them to a MSD clinic for support. First, tell me about the work you do in your home. What housework and childcare do you do?”

Encourage and prompt as required towards achieving a full answer:

- Go through the seven days if necessary
- Do you get any help? (From whom)
- What tasks are not getting done? (By participant? Not done at all?)

*Prompts to elaborate on primary question should be provided to collect information on:*

1. Biomechanical aspects

- How much physical effort does [specified tasks in turn] require?
- Which part of housework initiated the MSD problem?
- Which tasks cause pain? Where? (neck, back, shoulder, etc.) Describe in full, including if exacerbated by other tasks.
- Which tasks do you do differently? In what way? Has this helped?
- Has the pain / movement problem got worse over time? Elaborate.

1. Psychosocial aspects

- Describe on any social or physical support given. How did this arise? (Asked for or offered) Has it changed / escalated?
- When struggling to cope because of pain, how does this make you feel?
  - How do you respond? (Emotionally and practically)
- Does anyone expect you to cope (as before?) and put pressure on you to do tasks?
  - How does this make you feel?
  - How do you respond? (Emotionally and practically)
- Has your social life changed? (as a result of MSDs, e.g. tasks take longer, feel more tired)
  - In what way?
  - How does this make you feel?
  - How do you respond?

*Goal objectives point*

“Thank you answering in detail as you have. We want to know how to support you do your housework without pain or distress. So, finally, can I ask:

- What (or who) do you rely on more than anything else to cope with your [MSD issue] as a housewife?”

Repeat thanks, and you will be back in touch with feedback.
